# Effect of fermented red ginseng on gut microbiota dysbiosis- or immobilization stress-induced anxiety, depression, and colitis in mice

**DOI:** 10.1016/j.jgr.2022.08.004

**Published:** 2022-08-19

**Authors:** Yoon-Jung Shin, Dong-Yun Lee, Joo Yun Kim, Keon Heo, Jae-Jung Shim, Jung-Lyoul Lee, Dong-Hyun Kim

**Affiliations:** a Neurobiota Research Center, College of Pharmacy, Kyung Hee University, Seoul, Republic of Korea; b R&BD Center, hy Co.Ltd., Yongin, Republic of Korea

**Keywords:** red ginseng, fermentation, depression, ginsenoside Rd, gut microbiota, AD, anxiety/depression, BDNF, brain-derived neurotropic factor, CK, 20(S)-β-D-glucopyranosyl protopanaxadiol, ELISA, enzyme-linked immunoassay, EPMT, elevated plus maze task, FMT, fecal microbiota transplantation, fRG, fermented red ginseng, FST, forced swimming test, HPA, hypothalamic–pituitary–adrenal, IL, interleukin, IS, immobilization stress, LDTT, light/dark transition task, RG, red ginseng, TNBS, 2,4,6-trinitrobenzenesulfonic acid, TST, tail suspension test, UCD, ulcerative colitis and depression, UCDF, the feces of patients with ulcerative colitis and depression, TNF, tumor necrosis factor

## Abstract

**Background:**

Red ginseng (RG) alleviates psychiatric disorders. Fermented red ginseng (fRG) alleviates stress-induced gut inflammation. Gut dysbiosis causes psychiatric disorders with gut inflammation. To understand the gut microbiota-mediated action mechanism of RG and fRG against anxiety/depression (AD), we investigated the effects of RG, fRG, ginsenoside Rd, and 20(S)-β-D-glucopyranosyl protopanaxadiol (CK) on gut microbiota dysbiosis-induced AD and colitis in mice.

**Methods:**

Mice with AD and colitis were prepared by exposing to immobilization stress (IS) or transplanting the feces of patients with ulcerative colitis and depression (UCDF). AD-like behaviors were measured in the elevated plus maze, light/dark transition, forced swimming, and tail suspension tests.

**Results:**

Oral gavage of UCDF increased AD-like behaviors and induced neuroinflammation, gastrointestinal inflammation, and gut microbiota fluctuation in mice. Oral administration of fRG or RG treatment reduced UCDF-induced AD-like behaviors, hippocampal and hypothalamic IL-6 expression, and blood corticosterone level, whereas UCDF-suppressed hippocampal BDNF^+^NeuN^+^ cell population and dopamine and hypothalamic serotonin levels increased. Furthermore, their treatments suppressed UCDF-induced colonic inflammation and partially restored UCDF-induced gut microbiota fluctuation. Oral administration of fRG, RG, Rd, or CK also decreased IS-induced AD-like behaviors, blood IL-6 and corticosterone and colonic IL-6 and TNF-α levels, and gut dysbiosis, while IS-suppressed hypothalamic dopamine and serotonin levels increased.

**Conclusion:**

Oral gavage of UCDF caused AD, neuroinflammation, and gastrointestinal inflammation in mice. fRG mitigated AD and colitis in UCDF-exposed mice by the regulation of the microbiota-gut-brain axis and IS-exposed mice by the regulation of the hypothalamic-pituitary-adrenal axis.

## Graphical abstract

**Figure:**
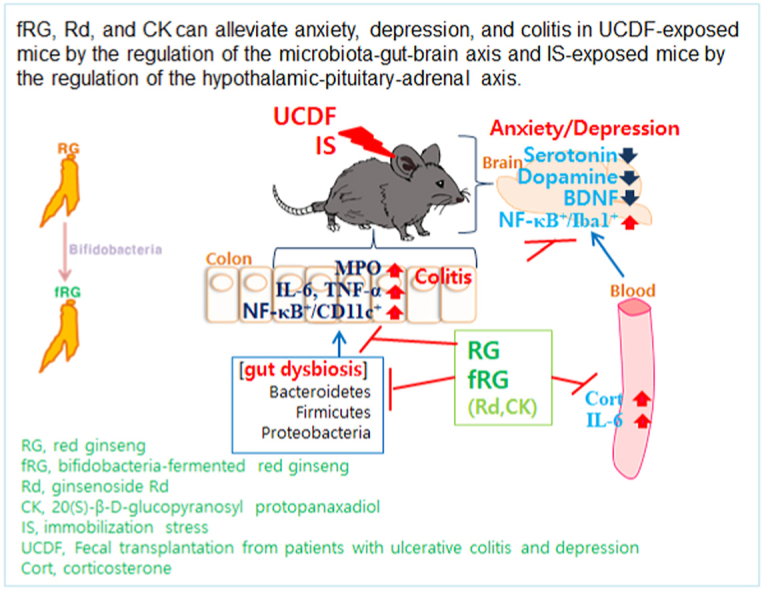

## 1 Introduction

Stress is a common experience in everyday life, and all organisms have evolved ways to cope with it [1]. Excessive exposure to stressors triggers the release of adrenaline and cortisol in the adrenal glands and inflammation-involved cytokines in the immune cells through the hypothalamic–pituitary–adrenal (HPA) axis activation [[2], [3], [4]]. The imbalanced activation of neuron-endocrine and hormonal function can give rise to affective disorders such as depression by suppressing brain-derived neurotrophic factor (BDNF), dopamine, and serotonin levels in the brain and gut inflammation and microbiota dysbiosis by inducing inflammatory immune responses in the gastrointestinal tract [[5], [6], [7]]. Gut microbiota dysbiosis causes gastrointestinal inflammation, which stimulate the translocation of gut microbiota byproducts such as endotoxin into the blood, leading to the occurrence of anxiety/depression (AD) with neuroinflammation [[8], [9], [10]]. For instance, immobilization stress (IS) causes anxiety, gastrointestinal inflammation, and overgrowth of Proteobacteria including *Escherichia coli* in mice [11]. The gavage of *Escherichia coli* also induces anxiety and gastrointestinal inflammation in mice [11,12]. These findings suggest that gut microbiota bidirectionally communicate with the brain via the regulation of the HPA and microbiota-gut-brain (MGB) axes.

Red ginseng (RG, prepared by steaming and drying the root of *Panax ginseng* Meyer, family Araliaceae) exhibits immunomodulatory, antidiabetic, antitumor, and anti-psychiatric effects [[13], [14], [15]]. Many studies suggest that the bioactive constituents of RG are ginsenosides [16,17]. These ginsenosides are transformed into absorbable compounds such as ginsenoside Rd, Rh2, and 20(S)-β-D-glucopyranosyl protopanaxadiol (CK) in the intestine of mice orally administered RG [[18], [19], [20]]. These metabolites exhibit more potent pharmacological activities than their parental ginsenosides such as ginsenosides Rb1 and Rg3 [[21], [22], [23]]. Therefore, to enhance the biological activity of RG, many processing skills have been developed [24,25]. Of these, fermentation increases the efficacy of RG against gut inflammation: fermented RG (fRG) mitigates 2,4,6-trinitrobenzenesulfonic acid (TNBS)- or *Escherichia coli*-induced colitis [26,27]. Furthermore, fRG and its constituent ginsenoside Rd ameliorate AD in IS-exposed mice [27]. Nevertheless, gut microbiota-mediated anxiolytic and anti-depressive action mechanism of fRG is still elusive.

Therefore, to understand gut microbiota-mediated anxiolytic and anti-depressive action mechanism of fRG, we prepared mice with AD and colitis by transplanting the feces of patients with ulcerative colitis and depression (UCDF) or exposing IS and examined the anxiolytic, anti-depressive, and anti-colitic effects of fRG.

## 2 Materials and methods

### 2.1 Preparation of RG and fRG

Water and ethanol extracts of red ginseng were prepared according to the method of Kim et al [27]. For the preparation of fRG, red ginseng ethanol extract was fermented by *Bifidobacterium adolescentis* HY8502 and *Bifidobacterium animalis* ssp. *lactis* HY8002 (36°C, 24 h), heated at 100°C for 15 min, and concentrated by evaporation (processed in hy, Seoul, Korea), as previously reported [27]. The ginsenoside Rb1, Rd, Rg3, and CK contents of RG and fRG were 3.2, 0.7, 2.1, and 2.5 mg/g. respectively, and 9.3, 4.1, 1.7, and 3.0 mg/kg, respectively.

### 2.2 Volunteers

As previously reported [28], patients with ulcerative colitis and depression (UCD) for the stool collection were recruited from Kyung Hee University Medical School (KHUMS; Seoul, Korea) in accordance to the protocol and informed consent forms approved by the KHUMS Clinical Study Care and Use Committee (IRB file No., 2018-12-004-003). All experiments were performed in compliance with the principles of the Helsinki Declaration and Korean Good Clinical Practice Guidelines.

### 2.3 Animals

C57BL/6 male mice (6-weeks old, 18––21 g) were purchased from Koatech Inc. (Korea). The mice were housed in plastic cages with a 5 cm-raised wire floor under a controlled conditions: the temperature, humidity, and light/dark cycle were 20^○^C–22^○^C, 50% ± 10%, and 12 h, respectively. Mice fed standard lab chow and water *ad libitum*. Approval for animal experiments was given by the Kyung Hee University Institutional Animal Care and Use Committee (IACUC No., KUASP(SE)-21098) and conducted according to the University Guide for Laboratory Animal Care and Usage.

Mice with AD and colitis were prepared by exposing to IS or transplanting UCDF, as previously reported [[28], [29], [30]].

First, to determine the effective dose of fRG against AD, we investigated the effects of RG and fRG against IS-induced AD in mice. Mice were categorized into six (NC, IS, IR50, IF25, IF50, and IF100) groups consisting of seven mice in each group. Mice of five (IS, IR50, IF25, IF50, and IF100) groups were exposed to IS (2 h/day) daily for 5 days. Test agents (IS, saline [vehicle]; IR50, 50 mg/kg/day RG extract; IF25, 25 mg/kg/day of fRG; IF50, 50 mg/kg/day of fRG; and IF100, 100 mg/kg/day of fRG, dissolved in saline) were orally gavaged daily for 5 days starting from 20 h after the final IS treatment. Mice of normal control group (NC) were treated with vehicle instead of test agents. AD-like behaviors were monitored 24 h after the final treatment with test agents.

Second, we investigated the effects of fRG constituents ginsenoside Rd and CK (Embo Laboratory, Daejeon, Korea) on IS-induced AD in mice. Mice were categorized into six (NC, IS, IR1, IR5, IC1, and IC5) groups consisting of seven mice in each group. Mice of five (IS, IR1, IR5, IC1, and IC5) groups were treated to IS daily for 5 days. Test agents (IS, saline [vehicle]; IR1, 1 mg/kg/day ginsenoside Rd; IR5, 5 mg/kg/day of ginsenoside Rd; IC1, 1 mg/kg/day of CK; and IC5, 5 mg/kg/day of CK, dissolved in saline) were orally gavaged daily for 5 days starting from 20 h after the final IS treatment. NC was treated with vehicle instead of test agents. AD-like behaviors were measured 24 h after the final treatment with test agents.

Third, to understand the MGB axis-mediated effect of fRG against AD in vivo, we examined the effects of RG, fRG, and their constituents against gut microbiota dysbiosis-induced colitis and AD*.* Mice were categorized into seven (NC, FT, FR50, FF25, FF50, FD1, and FC1) groups consisting of seven mice in each group. UCDF (10 mg/kg/day) was orally transplanted into mice of six (FT, FR50, FF25, FF50, FD1, and FC1) groups daily for 5 days. Test agents (FT, saline [vehicle]; FR50, 50 mg/kg/day RG extract; FF25, 25 mg/kg/day of fRG; FF50, 50 mg/kg/day of fRG; FR1, 1 mg/kg/day ginsenoside Rd; and FC1, 1 mg/kg/day of CK, dissolved in saline) were orally gavaged daily for 5 day after the final UCDF treatment. NC group was treated with vehicle instead of UCDF and test agents. AD-like behaviors were observed 24 h after the final treatment with test agents.

For the bacterial suspension for FMT, UCDFs were freshly and sterilely collected, suspended in sterilized saline at 4^o^C, and performed the centrifugation (500 *g,* 5 min). The supernatant was centrifuged (5000 *g,* 4^o^C, 5 min). The precipitate was suspended in saline. The fecal precipitate suspension were used for FMT experiments.

Mice were sacrificed 20 h after the final behavior tasks. Blood, colons, and brains were then removed and stored at −80 °C for the further study.

### 2.4 Behavioral tasks

The elevated plus maze task (EPMT), light/dark transition task (LDTT), forced swimming test (FST), and tail suspension test (TST) were carried out in accordance with the method of Jang et al [9,28].

### 2.5 ELISA

Tumor necrosis factor (TNF)-α, interleukin (IL)-1β, IL-6, myeloperoxidase, corticosterone, dopamine, and serotonin levels were determined using ELISA kits, as previously reported [9].

### 2.6 Immunofluorescence staining

Immunofluorescence staining for NF-κB^+^Iba1^+^, BDNF^+^NeuN^+^, and NF-κB^+^CD11c^+^ cells in the hippocampus, hypothalamus, and colon tissues was performed according to the method of Lee et al [30]. The stained samples were photographed with a confocal microscope.

### 2.7 Gut microbiota composition assay

The bacterial DNA was extracted from fresh mouse feces using a QIAamp DNA stool mini kit (Qiagen, Hilden, Germany) and amplified using barcoded primers targeted a bacterial 16S rRNA V4 region gene [12]. Amplicons were sequenced using Illumina iSeq100 (San Diego, CA). Microbiota data were deposited in the NCBI's short read archive (PRJNA741596).

### 2.8 Statistical analysis

All data values are described as mean ± standard deviation (SD). The significance was analyzed by one way analysis of variance followed by a Duncan multiple range test (P < 0.05) using GraphPad Prism 9.

## 3 Results

### 3.1 fRG mitigated IS-induced AD, colitis, and gut microbiota dysbiosis in mice

**Fig. 1:**
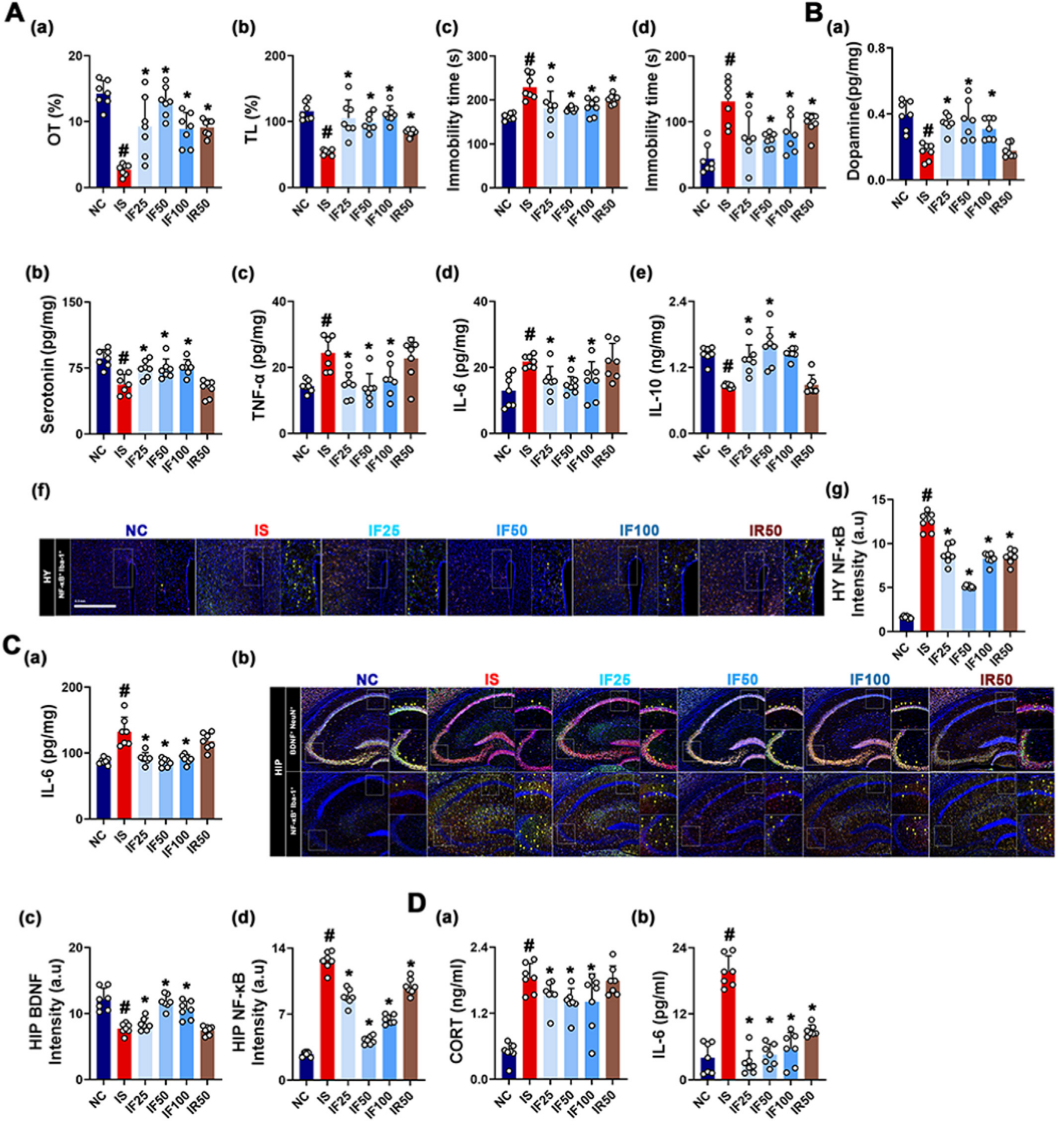
RG and fRG mitigated IS-induced AD in mice. (A) Effects on AD-like behaviors: OT in the EPMT (a), LT in the LDTT (b), and immobility time in the TST (c) and FST (d). (B) Effects on dopamine (a), serotonin (b), TNF-α (c), IL-6 (d), IL-10 (e) levels, NF-κB^+^Iba1^+^ cell population (f), and intensity of (f) (g) in the hypothalamus. (C) Effects on IL-6 expression (a), NF-κB^+^Iba1^+^ and BDNF^+^NeuN^+^ cell populations (b), and (b) intensities (c, d) in the hippocampus. (D) Effects on corticosterone (CORT, a) and IL-6 levels (b) in the blood. Mice were exposed to IS and test agents (IS, vehicle [saline]; IF50, 25 mg/kg/day of fRG; IF50, 50 mg/kg of fRG; IF100, 100 mg/kg/day of fRG; and IR50, 50 mg/kg/day of RG) were gavaged in IS-treated mice. Normal control group (NC) was treated with saline. Data values are represented as mean ± SD (n = 7). ^#^p < 0.05 vs. NC. ∗p < 0.05 vs. IS.

fRG ameliorates AD in IS-exposed mice [27]. Therefore, to confirm the anxiolytic and anti-depressive effect of fRG and determine its effective dosage, we investigated the effects of RG and fRG on IS-exposed AD and gut inflammation in mice. Exposure to IS increased AD-like behaviors in the EPMT, LDTT, TST, and FST and IL-6 expression and NF-κB^+^Iba1^+^ cell number in the hippocampus and hypothalamus, whereas BDNF^+^NeuN^+^ cell number in the hippocampus and serotonin and dopamine levels in the hypothalamus decreased (Fig. 1). However, oral administration of RG or fRG reduced IS-induced AD-like behaviors, hippocampal and hypothalamic IL-6 expression, and hippocampal NF-κB^+^Iba1^+^ cell number. Their treatments partially increased IS-induced suppression of hippocampal BDNF^+^NeuN^+^ cell number and hypothalamic serotonin and dopamine levels. fRG at a dose of 50 mg/kg was the most effective.

**Fig. 2:**
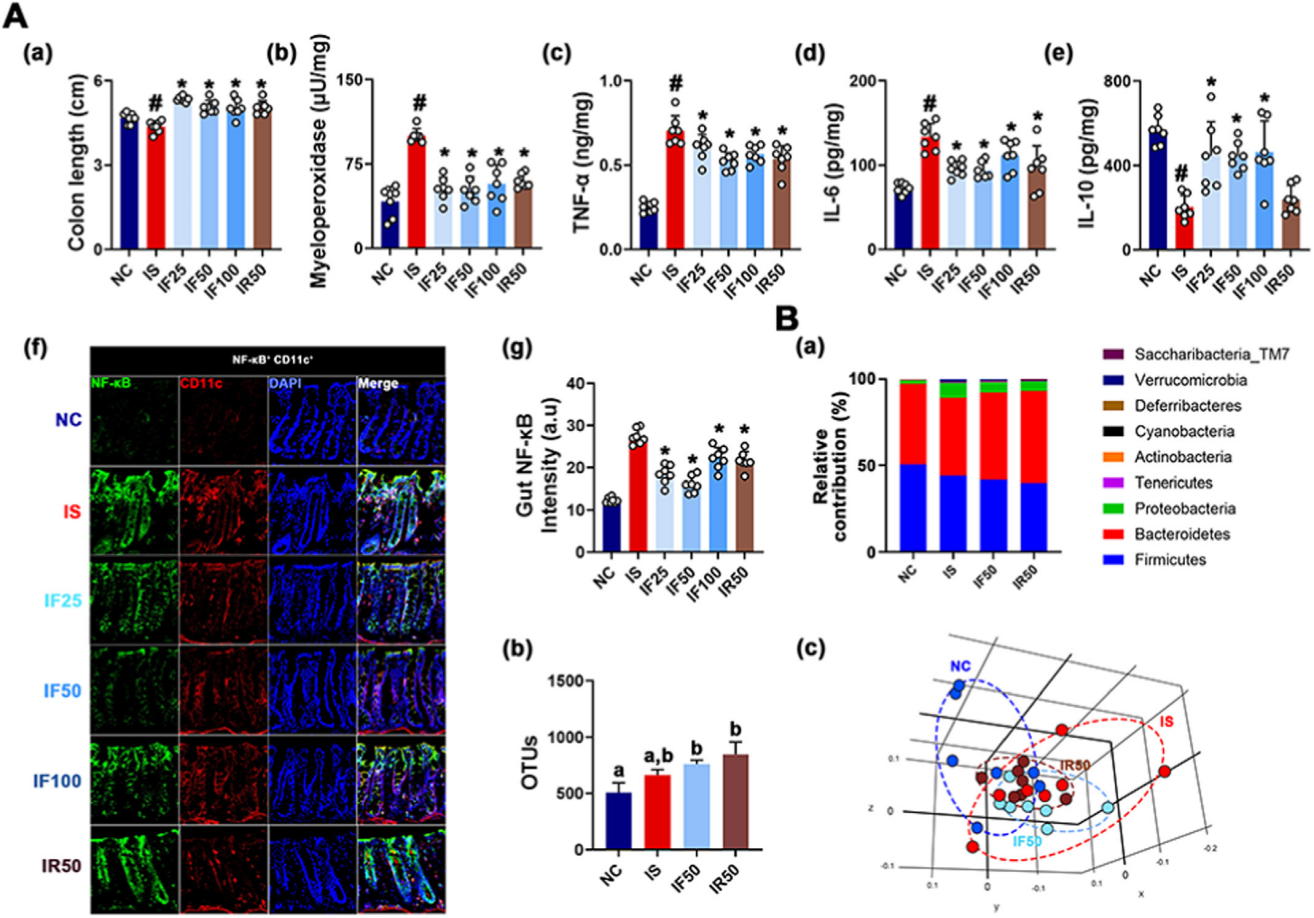
RG and fRG mitigated IS-induced colitis in mice. (A) Effects on colitis: colon length (a), myeloperoxidase (b), TNF-α (c), IL-6 (d), and IL-10 expression (e), NF-κB^+^CD11c^+^ cell population (f), and (f) intensity (g) in the colon. (B) Effect on fecal microbiota composition, analyzed by the pyrosequencing: (a) phylum level, (b) OTU, and (c) principal coordinate analysis (PCoA) plot (b). Test agents were treated, as described in Fig. 1. Data values are represented as mean ± SD (n = 7). ^#^p < 0.05 vs. NC. ∗p < 0.05 vs. IS.

Exposure to IS also caused colitis in mice: it shortened colon and increased colonic myeloperoxidase, TNF-α, and IL-6 expression and NF-κB^+^CD11c^+^ cell (dendritic and macrophage cells) number, resulting in colonic inflammation. However, oral administration of RG or fRG down-regulated IS-induced myeloperoxidase, TNF-α, and IL-6 expression, and NF-κB^+^CD11c^+^ cell number (Fig. 2). Furthermore, IS exposure also affected the gut microbiota composition: it shifted β-diversity, not α-diversity (OTU richness). Furthermore, IS exposure increased Proteobacteria and Tenericutes populations, whereas Actinobacteria population decreased. However, RG or fRG treatment partially alleviated IS-induced gut microbota fluctuation. Their treatments decreased Proteobacteria population and increased Actinobacteria population in IS-treated mice.

### 3.2 Ginsenosides Rd and CK mitigated AD, colitis, and gut dysbiosis in IS-exposed mice

**Fig. 3:**
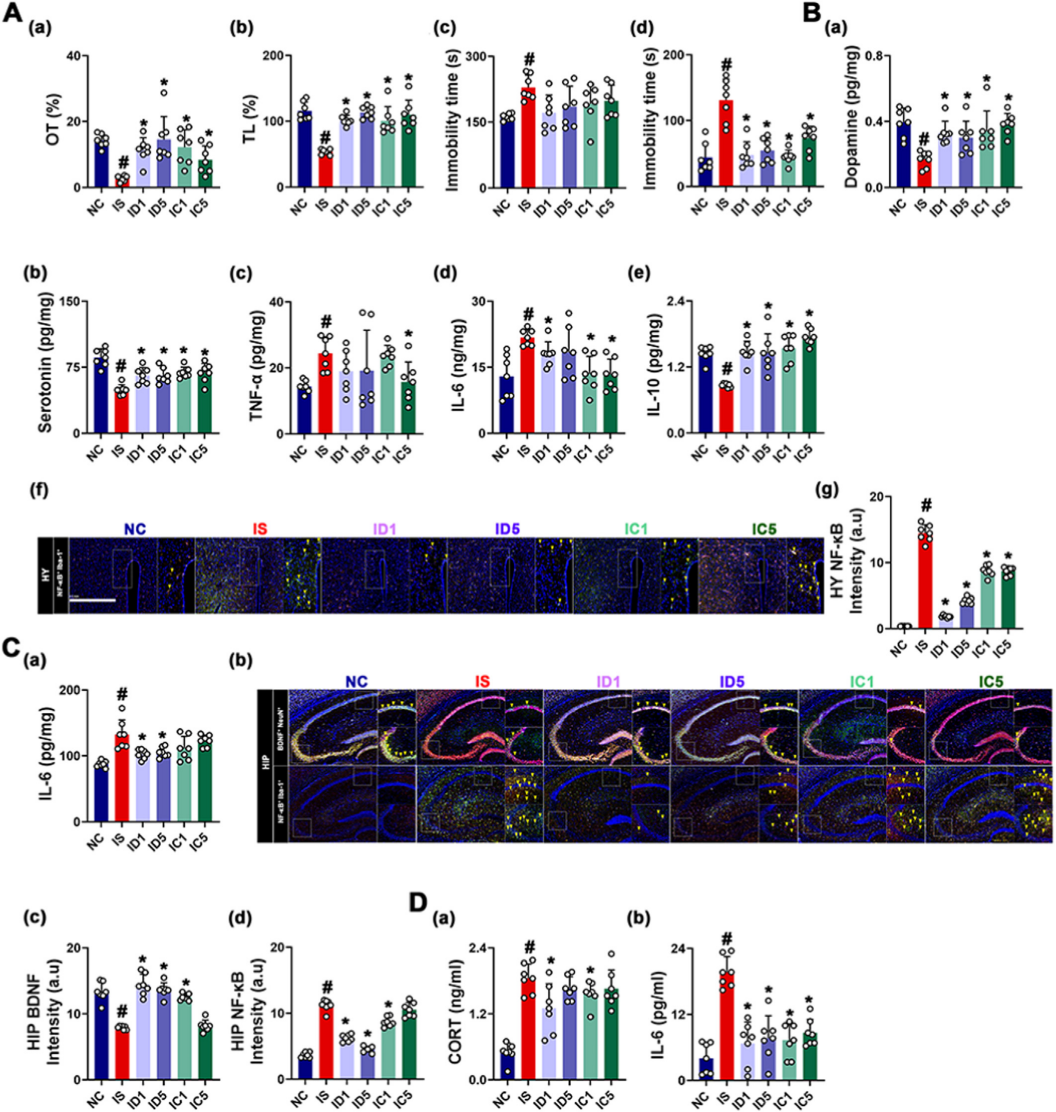
Ginsenoside Rd and compound K mitigated IS-induced AD in mice. (A) Effects on AD-like behaviors: OT in the EPMT (a), LT in the LDTT (b), and immobility time in the TST (c) and FST (d). (B) Effects on dopamine (a), serotonin (b), TNF-α (c), IL-6 (d), IL-10 (e) levels, NF-κB^+^Iba1^+^ cell population (f), and (f) intensity (g) in the hypothalamus. (C) Effects on IL-6 expression (a), NF-κB^+^Iba1^+^ and BDNF^+^NeuN^+^ cell populations (b), and (b) intensities (c, d) in the hippocampus. (D) Effects on corticosterone (CORT, a) and IL-6 levels (b) in the blood. Mice were exposed to IS and test agents (IS, vehicle [saline]; ID1, 1 mg/kg/day of ginsenoside Rd; ID5, 5 mg/kg of ginsenoside Rd; IC1, 1 mg/kg/day of CK; and IC5, 5 mg/kg/day of CK) were gavaged. Normal control group (NC) was treated with saline. Data values are represented as mean ± SD (n = 7). ^#^p < 0.05 vs. NC. ∗p < 0.05 vs. IS.

Next, we investigated the effects of ginsenoside Rd and CK on IS-induced AD and gut inflammation in mice. Treatment with ginsenoside Rd or CK at doses of 1 mg/kg and 5 mg/kg also suppressed IS-induced AD-like behaviors, hippocampal and hypothalamic IL-6 expression (Fig. 3). However, they increased IS-suppressed hypothalamic serotonin and dopamine levels. The difference between the efficacies of Rd and CK against AD and neuroinflammation was not significant.

**Fig. 4:**
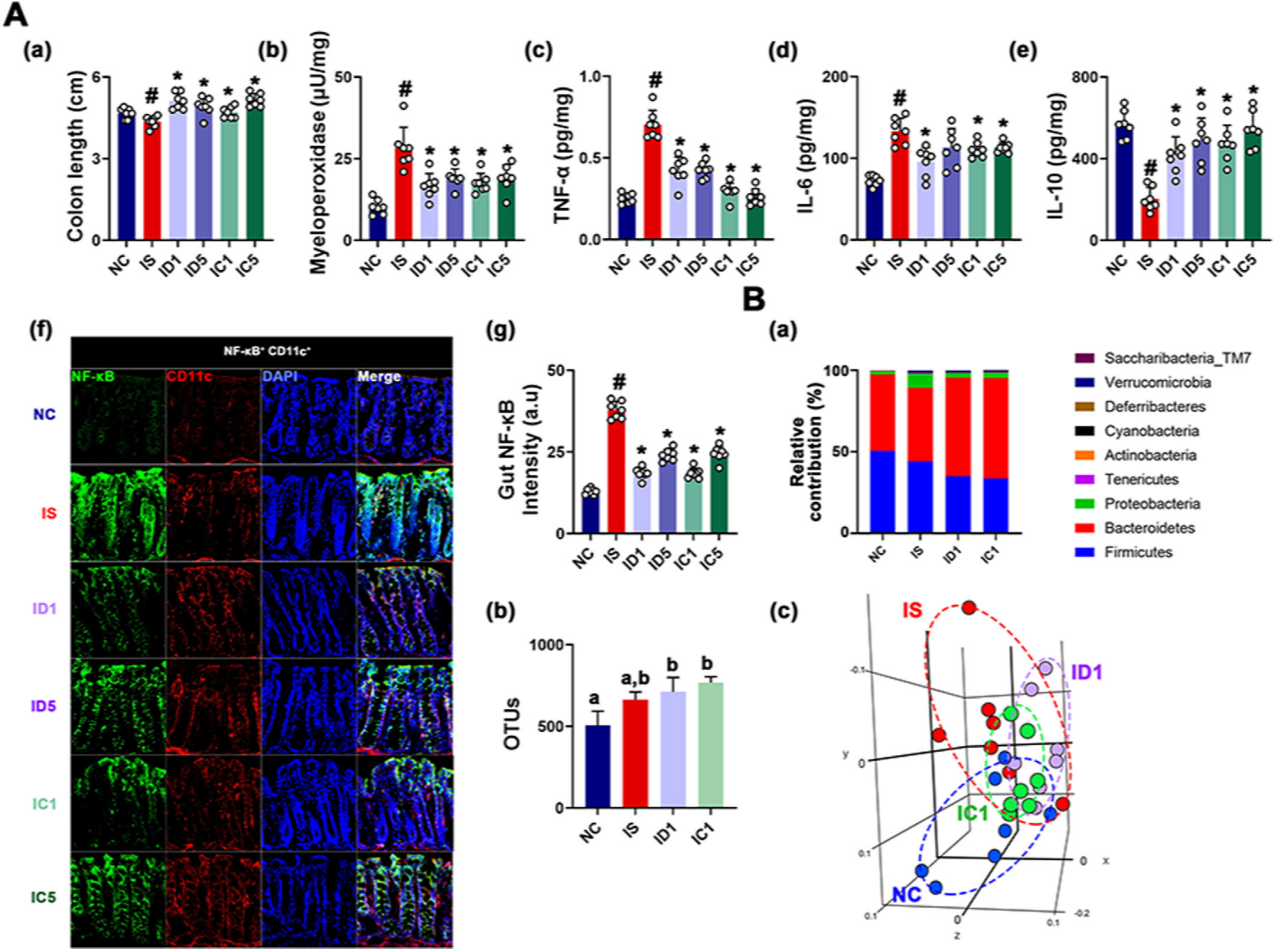
Ginsenoside Rd and compound K mitigated IS-induced colitis and gut micriboitoa fluctuation in mice. (A) Effects on colitis: colon length (a), myeloperoxidase (b), TNF-α (c), IL-6 (d), and IL-10 expression (e), NF-κB^+^CD11c^+^ cell population (f), and (f) intensity (g) in the colon. (B) Effect on the composition of gut microbiota, analyzed by the pyrosequencing: (a) phylum level, (b) OTU, and (c) principal coordinate analysis (PCoA) plot (b) based on Jensen-Shannon analysis. Test agents were treated, as described in Fig. 3. Data values are represented as mean ± SD (n = 7). ^#^p < 0.05 vs. NC. ∗p < 0.05 vs. IS.

Oral gavage of Rd or CK at doses of 1 mg/kg and 5 mg/kg also suppressed IS-induced colitis: their treatments alleviated colon shortening and down-regulated colonic myeloperoxidase, TNF-α, and IL-6 expression, and NF-κB^+^CD11c^+^ cell number (Fig. 4). The difference between the efficacies of Rd and CK against colitis was not significant. Oral gavage of ginsenoside Rd or CK modified IS-shifted gut microbiota composition: they increased α-diversity (OTU) and β-diversity (PCoA) and Bacteroidetes population, whereas IS-induced Proteobacteria number decreased.

### 3.3 fRG, ginsenosides Rd, and CK mitigated AD in mice transplanted with UCDF

**Fig. 5:**
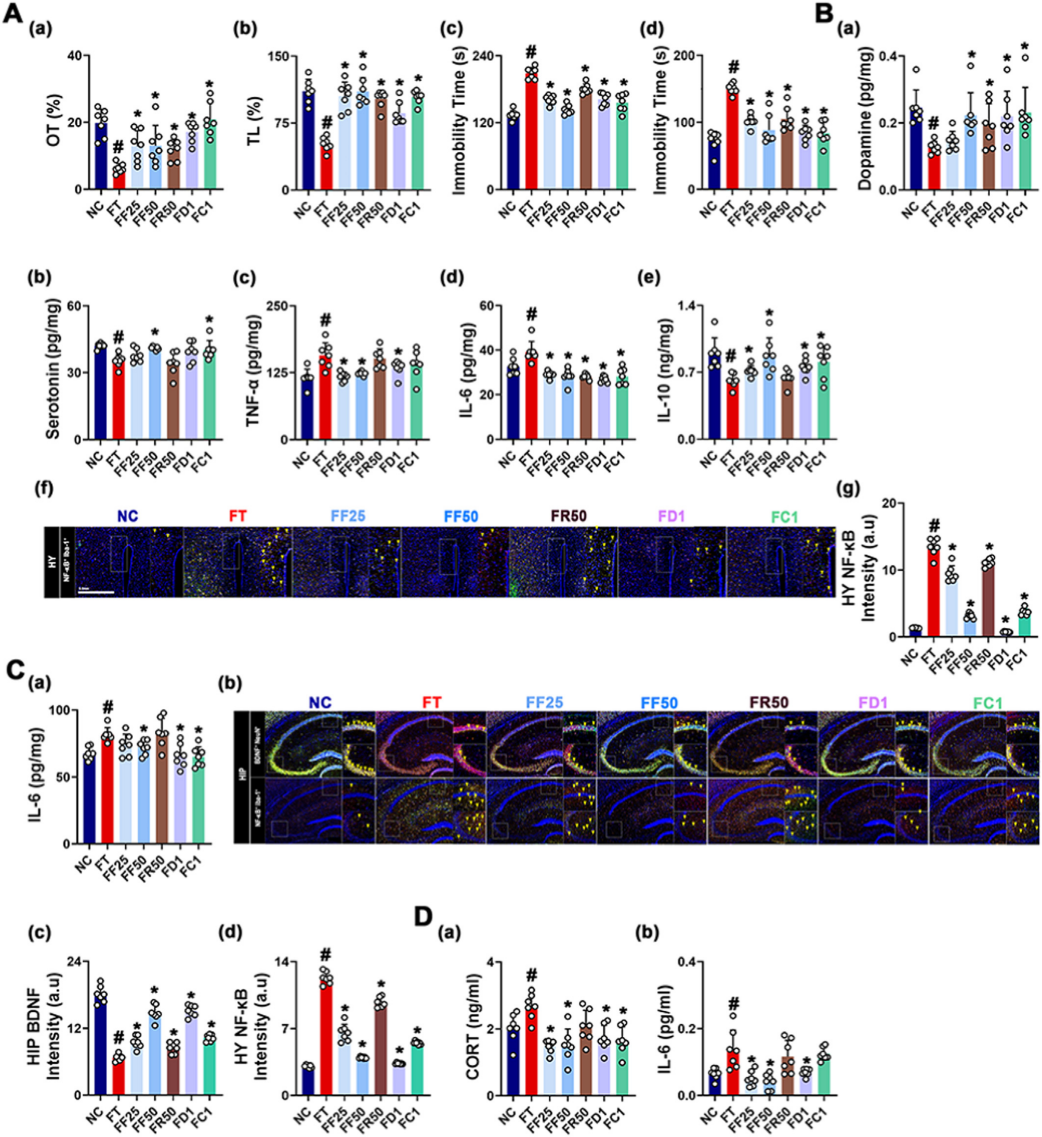
RG, fRG, ginsenoside Rd, and CK mitigated UCDF-induced AD in mice. (A) Effects on AD-like behaviors: OT in the EPMT (a), LT in the LDT (b), and immobility time in the TST (c) and FST (d). (B) Effects on dopamine (a), serotonin (b), TNF-α (c), IL-6 (d), IL-10 (e) levels, NF-κB^+^Iba1^+^ cell population (f), and (f) intensity (g) in the hypothalamus. (C) Effects on IL-6 expression (a), NF-κB^+^Iba1^+^ and BDNF^+^NeuN^+^ cell populations (b), and (b) intensities (c, d) in the hippocampus. (D) Effects on corticosterone (CORT, a) and IL-6 levels (b) in the blood. Mice were exposed to UCDF and test agents (FT, vehicle [saline]; FF25, 25 mg/kg/day of fRG; FF50, 50 mg/kg of fRG; FR50, 50 mg/kg/day of RG; ID1, 1 mg/kg/day of ginsenoside Rd; and IC1, 1 mg/kg/day of CK) were gavaged. The normal control group (NC) was treated with saline. Data values are represented as mean ± SD (n = 7). ^#^p < 0.05 vs. NC. ∗p < 0.05 vs. FT.

Gut dysbiosis is in close connection with the outbreak of psychiatric disorder [31,32]. Oral transplantation of UCDF causes AD and colonic inflammation in mice [28]. Therefore, to understand whether fRG could alleviate gut microbiota dysbiosis-induced AD and gut inflammation in vivo, we transplanted the feces from patients with UCD into mice and investigated the effects of RG and fRG on AD in the UCDF-transplanted mice (Fig. 5). Oral gavage of UCDF increased AD-like behaviors in the EPMT to 45.1% of NC group, LDTT to 32.2% of NC, TST to 39.7% of NC, and FST to 192.4% of NC, respectively. And UCDF treatment also induced hippocampal and hypothalamic IL-6 expression and NF-κB^+^Iba1^+^ cell number, while hippocampal BDNF^+^NeuN^+^ cell number and hypothalamic serotonin and dopamine levels decreased. However, oral administration of RG and fRG recovered UCDF-suppressed OT in the EPMT to 98.6% and 90.5% of NC, respectively, and UCDF-suppressed LT in the LDTT to 65.2% and 59.6% of NC, respectively, and UCDF-induced immobility time in the TST to 133.6% and 201.7% of NC, respectively, and in the FST to 122.8% and 147.2% of mice treated with UCDF alone, leading to an decrease in AD-like behaviors. And their treatments also suppressed UCDF-induced hippocampal and hypothalamic IL-6 expression and NF-κB^+^Iba1^+^ cell number and increased UCDF-suppressed hippocampal BDNF^+^NeuN^+^ cell number and hypothalamic serotonin and dopamine levels.

Furthermore, treatment with ginsenoside Rd or CK suppressed UCDF-induced AD-like behaviors, hippocampal and hypothalamic IL-6 expression and NF-κB^+^Iba1^+^ cell number. Their treatments increased IS-suppressed hypothalamic serotonin and dopamine levels. The difference between the efficacies of Rd and CK against AD and neuroinflammation was not significant.

### 3.4 fRG, ginsenosides Rd, and CK mitigated colitis and gut microbiota fluctuation in mice transplanted with UCDF

**Fig. 6:**
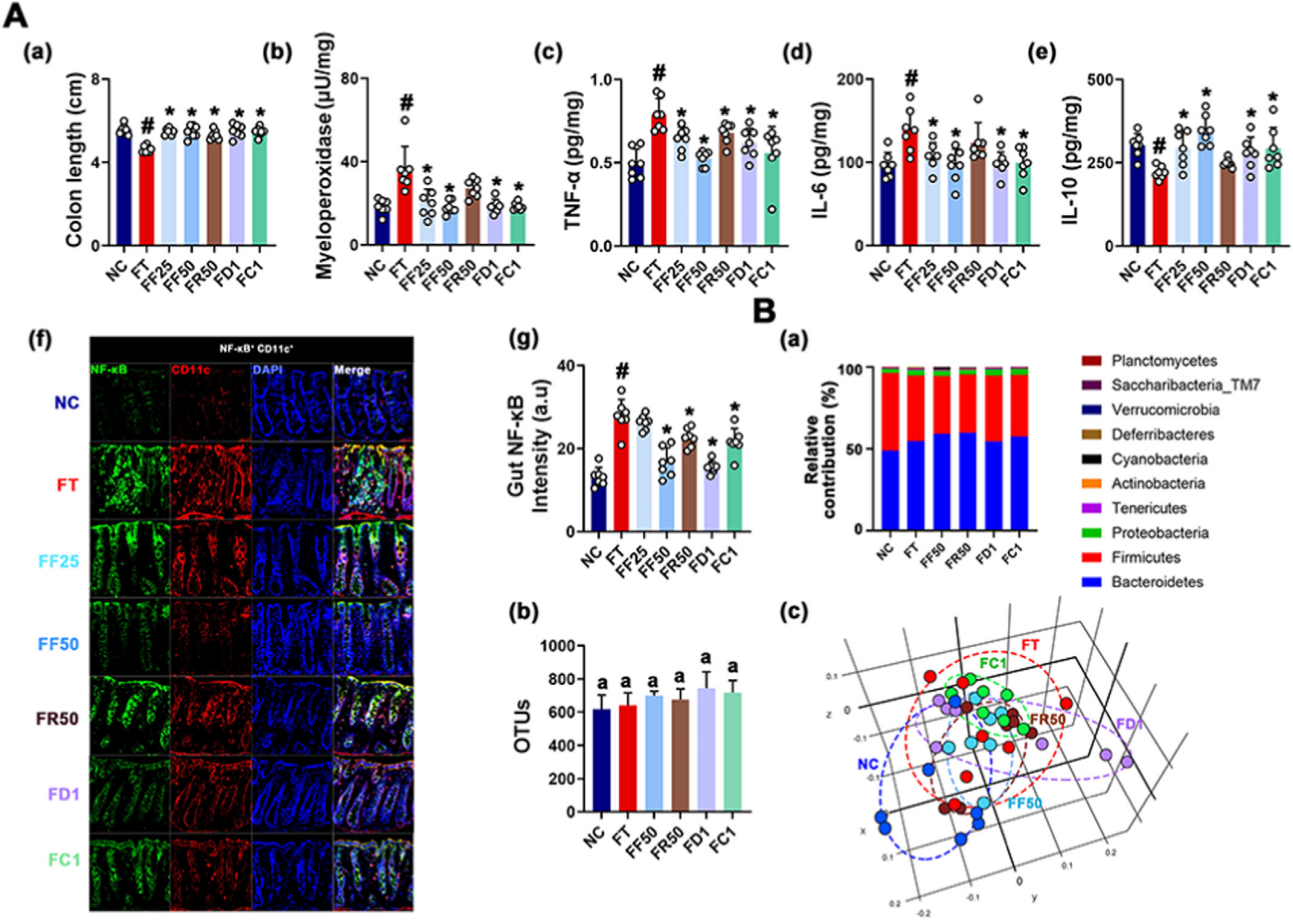
RG, fRG, ginsenoside Rd, and CK mitigated UCDF-induced colitis and gut microbiota fluctuation in mice. (A) Effects on colitis: colon length (a), myeloperoxidase activity (b), TNF-α (c), IL-6 (d), and IL-10 expression (e), NF-κB^+^CD11c^+^ cell population (f), and its intensity (g) in the colon. (B) Effect on the composition of gut microbiota, analyzed by the pyrosequencing: (a) phylum level, (b) OTU, and (c) principal coordinate analysis (PCoA) plot (b) based on Jensen-Shannon analysis. Test agents were treated, as described in Fig. 5. The normal control group (NC) was treated with saline. Data values are represented as mean ± SD (n = 7). ^#^p < 0.05 vs. NC. ∗p < 0.05 vs. FT.

The effects of fRG, RG, ginsenoside Rd, and CK on UCDF-induced colitis were investigated in mice. Oral gavage of UCDF shortened colon and upregulated myeloperoxidase, TNF-α, and IL-6 expression, and NF-κB^+^CD11c^+^ cell number (Fig. 6). However, treatment with RG or fRG suppressed UCDF-induced myeloperoxidase, TNF-α, and IL-6 expression, and NF-κB^+^CD11c^+^ cell number, leading to the amelioration of colitis.

UCDF treatment shifted gut bacterial β-diversity, whereas the difference of α-diversity was not significant. UCDF exposure increased Enterobacteriaceae population, while Bifidobacterium spp. population decreased. However, RG or fRG treatment decreased UCDF-induced Enterobacteriaceae population, while Actinobacteria population increased.

Oral gavage of ginsenoside Rd or CK at a dose of 1 mg/kg also alleviated UCDF-induced NF-κB/CD11c^+^ cell number and TNF-α, IL-6, and myeloperoxidase expression. The difference between the efficacies of Rd and CK against colitis was not significant. Oral gavage of ginsenoside Rd or CK also modified IS-shifted gut microbiota composition: they increased α-diversity (OTU), β-diversity (PCoA), and Bacteroidetes population at the phylum level, while IS-induced Proteobacteria population decreased.

## 4 Discussion

Exposure to stressors causes AD by inducing the release of adrenal hormones and proinflammatory cytokines through activation of HPA axis [2,3,31]. In the present study, exposure to IS increased AD-like behaviors. IS exposure also suppressed hypothalamic dopamine and serotonin levels and hippocampal BDNF expression and increased hippocampal and hypothalamic TNF-α and IL-6 expression, and blood IL-6 and corticosterone levels, leading to neuroinflammation. Moreover, it caused colitis and altered gut microbiota composition. These observations suggest that IS can cause AD, neuroinflammation, colitis, and gut microbiota dysbiosis by imbalanced activation of the HPA axis.

Oral gavage of antibacterials such as ampicillin and fecal microbiota transplantation from mice or patients with colonic inflammation cause gut dysbiosis, colitis, and neuroinflammation in mice, leading to AD [9,28]. In the present study, we also found that oral transplantation of UCDF increased AD-like behaviors, neuroinflammation, colitis, and gut microbiota fluctuation. Furthermore, UCDF treatment increased blood IL-6 and corticosterone levels as well as hippocampal and hypothalamic TNF-α and IL-6 expression and NF-κB^+^Iba1^+^ cell numbers and decreased hypothalamic dopamine and serotonin levels. Jang et al observed that the induction of gastrointestinal inflammation by TNBS, a colitis inducer, caused neuroinflammation in mice [8]. These findings make a suggestion that gut microbiota dysbiosis can raise AD with systemic inflammation by imbalanced activation of the MGB axis.

We found that fRG, Rd, and CK alleviated AD-like behaviors and colitis in mice with gut microbiota dysbiosis-induced AD and gut inflammation. Furthermore, fRG, Rd, and CK partially restored UCDF-induced gut microbiota fluctuation. Treatment with fRG, Rd, or CK also reduced UCDF-induced blood corticosterone and IL-6 levels and hippocampal and hypothalamic TNF-α and IL-6 expression and NF-κB^+^/Iba1^+^ cell number. Treatment with fRG, Rd, or CK increased UCDF-suppressed hypothalamic dopamine and serotonin levels and hippocampal BDNF expression. These findings suggest that fRG, Rd, and CK may alleviate AD with colitis through the modulation of gut microbiota.

We also found that fRG, Rd, and CK alleviated IS-suppressed AD-like behavior and colitis in mice. Furthermore, fRG, Rd, and CK increased hypothalamic dopamine and serotonin levels and hippocampal BDNF expression and decreased hypothalamic and hippocampal TNF-α and IL-6 expression and NF-κB^+^Iba1^+^ cell numbers, and blood corticosterone and IL-6 levels. Furthermore, their treatments partially restored IS-induced gut microbiota fluctuation. In particular, they reduced fecal Proteobacteria population. Tode et al observed that RG alleviated psychiatric symptoms in patients with menopausal syndrome [33]. Han et al reported that fluoxetine, an antidepressant, alleviated AD, colitis, and gut dysbiosis [34]. These results make a suggestion that fRG, Rd, and CK can alleviate AD by the regulation of HPA axis activation and can improve colitis and gut dysbiosis.

Although stressors more exaggeratedly cause AD in germ-free mice than in SPF mice, the susceptibility of germ-free mice to the stressors was attenuated by the FMT from SPF mice [35,36]. Jang et al observed that *Lactobacillus johnsonii* alleviated TNBS-induced neuroinflammation in mice [8]. The disruption of gut microbiota by ampicillin treatment raised AD in mice through colitis and neuroinflammation [9]. However, FMT or some probiotics, which relieve stressors-induced gut dysbiosis, alleviate colitis, leading to the decrease in AD [28,29]. These findings suggest that RG, fRG, Rd, and CK may alleviate gut microbiota dysbiosis-induced AD with neuroinflammation and gut inflammation of by the modulation of MGB axis activation.

In addition, when RG or fRG is orally given in humans and animals, their main components such as ginsenoside Rb1 and Rc are seldom absorbed into the body due to their hydrophilic and high weight molecules [13,18]. Therefore, these components get in touch with bacteria in the intestine and are able to be transformed into absorbable metabolites such as ginsenoside Rd and CK [13,18,37,38]. Based on these findings, we evaluated the effects of ginsenoside Rd and CK on UCDF- or IS-induced AD and colitis in mice. These ginsenosides potently alleviated AD and gastrointestinal inflammation. They also partially restored IS- or UCDF-induced gut microbiota fluctuation. The protective effect of RG against the cytotoxicity of neuron cells (SH-SY5Y cells) by hydrogen peroxide was more potent than that of fRG (Supplement Figure S1). However, the efficacy of Rd was not significantly different from that of CK in vitro. Recent studies have suggested that ginsenosides Rb1 and Rg3 and their metabolites, ginsenosides Rd, Rh2, and CK, can alleviate stressors-induced depression in rodents [[39], [40], [41]]. These observations make a suggestion that the efficacy of RG and fRG against AD and colitis can be dependent on their bioactive and absorbable ginsenosides. Moreover, their efficacies may be enhanced by the transformation of their ginsenosides into absorbable ginsenosides into the blood. This proposal is supported by a former report that Rd and CK were more completely absorbed into the bloods of volunteers and mice orally administered with fRG more highly than those orally treated with RG [42,43]. This finding suggests that the ginsenosides of fRG may be transformed into absorbable metabolites by gut micribota and thereby fRG may be beneficial for the treatment of AD and colitis.

In conclusion, IS caused AD by the imbalanced activation of the HPA axis, resulting in the colitis and gut dysbiosis. UCDF caused gut dysbiosis and colitis, leading to the outbreak of AD with neuroinflammation by imbalanced activation of the MGB axis. Fermentation of RG can enhance the efficacy of RG against AD and colitis: fRG and its constituents Rd and CK may mitigate AD and colitis in IS-exposed mice by the regulation of the HPA axis and in UCDF-exposed mice by the regulation of the MGB axis.

## Declaration of competing interest

The authors declare no conflict of interest.
