# Corrigendum to ‘Effect of fermented red ginseng on gut microbiota dysbiosis- or immobilization stress-induced anxiety, depression, and colitis in mice’[Journal of Ginseng Research Volume 47, Issue 2, March 2023, Pages 255-264]

**DOI:** 10.1016/j.jgr.2023.07.001

**Published:** 2023-07-04

**Authors:** Yoon-Jung Shin, Dong-Yun Lee, Joo Yun Kim, Keon Heo, Jae-Jung Shim, Jung-Lyoul Lee, Dong-Hyun Kim

**Affiliations:** a Neurobiota Research Center, College of Pharmacy, Kyung Hee University, Seoul, 130872, Republic of Korea; b R&BD Center, hy Co.Ltd., Yongin, Republic of Korea

The authors regret that on page 257 of the paper, " 2.8. All data values of Statistical analysis are described as mean ± standard deviation (SD). The significance was analyzed by one way analysis of variance followed by a Duncan multiple range test (P < 0.05) using GraphPad Prism9." Answers questions about The statistic is Dunn's multiple comparisons test, not the Duncan multiple range test, incorrectly spelled as Duncan multiple range test. So I'll correct it with Dunn's multiple comparisons test.

2.8. Statistical analysis

All data values are described as mean ± standard deviation (SD). The significance was analyzed by one way analysis of variance followed by a **Dunn's multiple comparisons test** (P < 0.05) using GraphPad Prism 9.

The authors would like to apologise for any inconvenience caused.

