# Supplementary material for: Effect of fermented red ginseng on gut microbiota dysbiosis- or immobilization stress-induced anxiety, depression, and colitis in mice
Source: J Ginseng Res. 2022 Aug 19;47(2):255–64. doi: 10.1016/j.jgr.2022.08.004 (PMC10014181; doi:10.1016/j.jgr.2022.08.004)
Supplement: Multimedia component 1 [file mmc1.docx]

**[Supplement]**

**Effect of fermented red ginseng on gut microbiota dysbiosis- or immobilization stress-induced anxiety, depression, and colitis in mice**


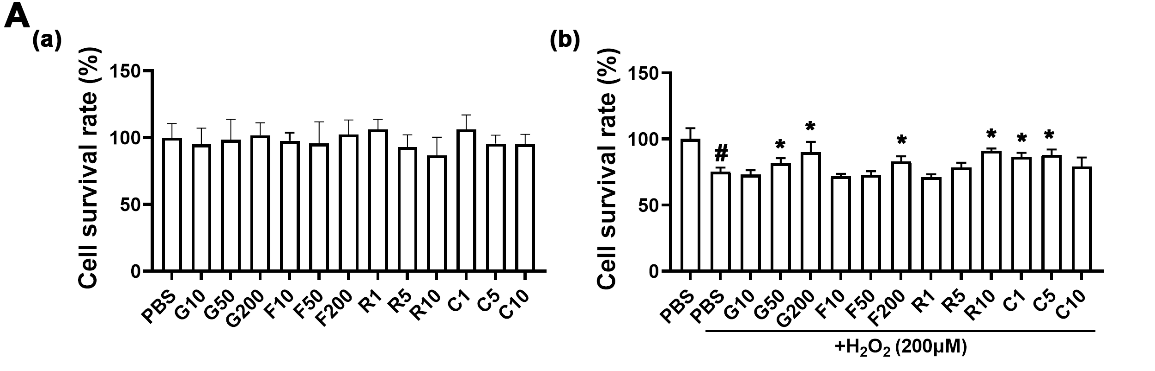


Figure S1. Effects of RG, fRG, ginsenoside Rd, and Compound K on the cell survival in SH-SY5Y cells treated with or without H2O2. Test agents (G10, 10 μg/mL of RG; G50, 50 μg/mL of RG; G200, 200 μg/mL of RG; F10, 10 μg/mL of fRG; F50, 50 μg/mL of fRG; F200, 200 μg/mL of fRG; R1, 1 μg/mL of gisnengoside Rd; R5, 5 μg/mL of ginsenoside Rd; R10, 10 μg/mL of ginsenoside Rd; C1, 1 μg/mL of compound K; C5, 5 μg/mL of compound K; C10, 10 μg/mL of compound K after treatment with LPS ( 100 ng/mL) in SH-SY5Y cells (1×10^6^ cell/mL).

Table S1. Effects of RG and fRG on the gut microbiota composition (at a phylum level) in mice treated with IS

|  | Composition (%) | | | |
| --- | --- | --- | --- | --- |
|  | NC | IS | FS50 | RS50 |
| Firmicutes | 50.7±10.2 | 44.0±12.9 | 41.7±8.6 | 39.9±10.6 |
| Bacteroidetes | 46.6±10.3 | 45.4±15.7 | 50.7±10.6 | 53.3±10.2 |
| Proteobacteria | 2.2±0.7 | 8.5±4.9***** | 5.8±2.6 | 5.5±2.4**^#^** |
| Tenericutes | 0.3±0.4 | 0.3±0.3 | 0.7±0.4 | 0.4±0.2 |
| Actinobacteria | 0.1±.0.1 | 0.1±0.0 | 0.1±0.1 | 0.1±0.1 |
| Cyanobacteria | 0.0±±0.0 | 0.1±0.1***** | 0.1±0.1 | 0.5±0.4 |
| Deferribacteres | 0.0±0.0 | 0.3±0.5 | 0.1±0.2 | 0.2±0.2 |
| Verrucomicrobia | 0.0±0.1 | 1.3±3.4 | 0.7±0.9 | 0.0±0.0 |
| Saccharibacteria_TM7 | 0.0±0.0 | 0.0±0.0 | 0.0±0.0 | 0.0±0.0 |

Values indicate mean± SD. ******p*<0.05 vs NC and **^#^***p*<0.05 vs IS.

Table S2. Effects of RG and fRG on the gut microbiota composition (at a family level) in mice treated with IS

|  | Composition (%) | | | |
| --- | --- | --- | --- | --- |
|  | NC | IS | FS50 | RS50 |
| Lactobacillaceae | 31.9±10.0 | 9.4±7.0***** | 4.8±4.2 | 0.8±0.4**^#^** |
| Muribaculaceae | 31.9±5.0 | 27.7±11.1 | 36.3±12.2 | 26.9±4.9 |
| Lachnospiraceae | 14.0±9.8 | 27.6±11.0***** | 28.4±9.3 | 30.4±10.3 |
| Prevotellaceae | 5.6±2.9 | 9.1±7.1 | 7.0±7.2 | 19.3±9.4**^#^** |
| Bacteroidaceae | 4.6±1.1 | 3.4±2.3 | 2.6±1.6 | 3.3±1.2 |
| Ruminococcaceae | 3.1±1.7 | 6.1±2.3 | 7.8±1.8 | 7.8±2.0 |
| Rikenellaceae | 2.9±1.4 | 3.7±2.6 | 3.2±2.1 | 2.5±1.4 |
| Helicobacteraceae | 1.8±0.7 | 7.2±4.9***** | 4.7±2.6 | 4.4±2.1 |
| Odoribacteraceae | 1.0±1.4 | 0.5±0.7 | 0.8±1.4 | 0.5±0.4 |
| Clostridiaceae | 0.9±1.2 | 0.1±0.1 | 0.1±0.1 | 0.1±0.0 |
| Desulfovibrionaceae | 0.3±0.3 | 1.0±0.7***** | 0.9±0.5 | 1.0±0.4 |
| Enterococcaceae | 0.3±0.2 | 0.1±0.1 | 0.1±0.1 | 0.0±0.0 |
| Porphyromonadaceae | 0.3±0.1 | 0.3±0.2 | 0.3±0.2 | 0.2±0.1 |
| AC160630_f | 0.3±0.1 | 0.5±0.4 | 0.3±0.4 | 0.4±0.2 |
| Christensenellaceae | 0.2±0.1 | 0.4±0.3 | 0.3±0.2 | 0.3±0.1 |
| Mycoplasmataceae | 0.2±0.4 | 0.2±0.3 | 0.5±0.4 | 0.3±0.1 |
| Dehalobacterium_f | 0.1±0.1 | 0.2±0.1 | 0.2±0.1 | 0.4±0.2 |
| Coriobacteriaceae | 0.1±0.1 | 0.1±0.0 | 0.1±0.1 | 0.1±0.1 |
| Erysipelotrichaceae | 0.1±0.1 | 0.0±0.0 | 0.1±0.0 | 0.1±0.0 |
| Sutterellaceae | 0.1±0.1 | 0.0±0.0 | 0.1±0.1 | 0.0±0.0 |
| Mogibacterium_f | 0.1±0.0 | 0.1±0.0 | 0.1±0.0 | 0.1±0.0 |
| PAC000197_f | 0.0±0.0 | 0.0±0.0 | 0.1±0.1 | 0.1±0.0 |
| FR888536_f | 0.0±0.0 | 0.1±0.1 | 0.1±0.1 | 0.5±0.4 |
| Deferribacteraceae | 0.0±0.0 | 0.3±0.5 | 0.1±0.2 | 0.2±0.2 |
| Akkermansiaceae | 0.0±0.1 | 1.3±3.4 | 0.7±0.9 | 0.0±0.0 |

Values indicate mean± SD. ******p*<0.05 vs NC and **^#^***p*<0.05 vs IS.

Table S3. Effects of RG and fRG on the gut microbiota composition (at a genus level) in mice treated with IS

|  | Composition (%) | | | |
| --- | --- | --- | --- | --- |
|  | NC | IS | FS50 | RS50 |
| Lactobacillus | 31.5±9.8 | 9.3±6.9***** | 4.8±4.2 | 0.8±0.4**^#^** |
| PAC000186_g | 10.2±2.8 | 7.53.4 | 10.0±3.8 | 7.2±2.6 |
| PAC001068_g | 7.5±2.2 | 6.9±3.1 | 8.0±2.9 | 6.7±1.2 |
| Bacteroides | 4.6±1.1 | 3.4±2.3 | 2.6±1.5 | 3.3±1.1 |
| KE159538_g | 4.2±3.5 | 5.0±6.6 | 2.2±1.6 | 2.0±1.0 |
| PAC001074_g | 2.9±2.4 | 1.6±1.3 | 1.5±0.9 | 0.7±0.5 |
| Prevotella | 2.5±1.6 | 2.3±2.2 | 2.0±2.2 | 4.9±2.5 |
| PAC000198_g | 2.3±0.4 | 2.8±0.9 | 3.5±0.4 | 2.5±0.7 |
| Alistipes | 2.3±1.5 | 3.3±2.3 | 2.7±2.1 | 2.1±1.3 |
| PAC001127_g | 1.9±0.8 | 1.3±0.9 | 2.2±0.9 | 2.0±0.5 |
| Helicobacter | 1.8±0.7 | 7.2±4.9***** | 4.7±2.6 | 4.4±2.1 |
| Alloprevotella | 1.4±2.1 | 1.5±1.3 | 0.5±0.5 | 0.0±0.0**^#^** |
| PAC001124_g | 1.2±1.7 | 0.9±0.9 | 1.3±2.1 | 0.9±0.9 |
| Muribaculum | 1.1±0.6 | 1.4±0.9 | 2.9±1.5**^#^** | 2.7±1.0**^#^** |
| PAC000664_g | 1.1±1.4 | 2.4±1.6 | 1.5±0.8 | 2.8±1.7 |
| PAC001066_g | 1.1±0.4 | 0.9±0.7 | 1.2±0.6 | 0.9±0.2 |
| Odoribacter | 1.0±1.4 | 0.5±0.7 | 0.8±1.4 | 0.5±0.3 |
| PAC001765_g | 1.0±0.6 | 1.1±0.4 | 1.1±0.3 | 0.8±0.2 |
| PAC001112_g | 0.9±0.3 | 1.6±1.3 | 0.9±0.4 | 1.1±0.4 |
| Paraprevotella | 0.9±0.9 | 2.3±2.6 | 0.9±1.3 | 0.7±0.7 |
| Arthromitus | 0.9±1.2 | 0.1±0.1 | 0.1±0.1 | 0.1±0.0 |
| Pseudoflavonifractor | 0.9±0.6 | 1.6±0.6***** | 2.2±0.7 | 2.0±0.6 |
| Prevotellaceae_uc | 0.9±0.7 | 3.1±4.9 | 3.6±5.2 | 13.7±7.0**^#^** |
| Oscillibacter | 0.7±0.8 | 2.3±1.3 | 2.0±0.9 | 2.5±0.9 |
| PAC001512_g | 0.7±0.6 | 1.0±0.6 | 2.9±1.6**^#^** | 0.6±0.2 |

Values indicate mean± SD. ******p*<0.05 vs NC and **^#^***p*<0.05 vs IS.

Table S4. Effects of Rd and CK on the gut microbiota composition (at a phylum level) in mice treated with IS

|  | Composition (%) | | | |
| --- | --- | --- | --- | --- |
|  | NC | IS | ID1 | IC1 |
| Firmicutes | 50.7±10.2 | 44.0±12.9 | 35.1±17.7 | 33.7±13.6 |
| Bacteroidetes | 46.6±10.3 | 45.4±15.7 | 60.4±19.4 | 61.6±14.9 |
| Proteobacteria | 2.2±0.7 | 8.5±4.9***** | 2.9±2.2 | 3.4±2.1**^#^** |
| Tenericutes | 0.3±0.4 | 0.3±0.3 | 0.2±0.1 | 0.3±0.1 |
| Actinobacteria | 0.1±.0.1 | 0.1±0.0 | 0.1±0.0 | 0.1±0.0 |
| Cyanobacteria | 0.0±±0.0 | 0.1±0.1***** | 0.2±0.2 | 0.6±0.6**^#^** |
| Deferribacteres | 0.0±0.0 | 0.3±0.5 | 0.0±0.0 | 0.0±0.0 |
| Verrucomicrobia | 0.0±0.1 | 1.3±3.4 | 1.1±1.0 | 0.3±0.2 |
| Saccharibacteria_TM7 | 0.0±0.0 | 0.0±0.0 | 0.0±0.0 | 0.0±0.0 |

Values indicate mean± SD. ******p*<0.05 vs NC and **^#^***p*<0.05 vs IS.

Table S5. Effects of Rd and CK on the gut microbiota composition (at a family level) in mice treated with IS

|  | Composition (%) | | | |
| --- | --- | --- | --- | --- |
|  | NC | IS | ID1 | IC1 |
| Lactobacillaceae | 31.9±10.0 | 9.4±7.0***** | 0.8±0.4**^#^** | 3.0±2.7**^#^** |
| Muribaculaceae | 31.9±5.0 | 27.7±11.1 | 35.6±9.3 | 38.1±12.6 |
| Lachnospiraceae | 14.0±9.8 | 27.6±11.0***** | 25.9±15.7 | 20.3±14.8 |
| Prevotellaceae | 5.6±2.9 | 9.1±7.1 | 15.4±8.2 | 17.0±10.3 |
| Bacteroidaceae | 4.6±1.1 | 3.4±2.3 | 5.9±3.8 | 4.0±2.7 |
| Ruminococcaceae | 3.1±1.7 | 6.1±2.3 | 7.6±3.4 | 9.6±1.0**^#^** |
| Rikenellaceae | 2.9±1.4 | 3.7±2.6 | 2.8±1.0 | 1.9±1.6 |
| Helicobacteraceae | 1.8±0.7 | 7.2±4.9***** | 1.9±1.8**^#^** | 2.3±1.9**^#^** |
| Odoribacteraceae | 1.0±1.4 | 0.5±0.7 | 0.1±0.1 | 0.0±0.0 |
| Clostridiaceae | 0.9±1.2 | 0.1±0.1 | 0.1±0.1 | 0.1±0.0 |
| Desulfovibrionaceae | 0.3±0.3 | 1.0±0.7***** | 0.9±1.2 | 0.4±0.2**^#^** |
| Enterococcaceae | 0.3±0.2 | 0.1±0.1 | 0.1±0.1 | 0.1±0.0 |
| Porphyromonadaceae | 0.3±0.1 | 0.3±0.2 | 0.4±0.1 | 0.2±0.0 |
| AC160630_f | 0.3±0.1 | 0.5±0.4 | 0.3±0.2 | 0.3±0.1 |
| Christensenellaceae | 0.2±0.1 | 0.4±0.3 | 0.3±0.2 | 0.3±0.1 |
| Mycoplasmataceae | 0.2±0.4 | 0.2±0.3 | 0.0±0.0 | 0.0±0.0 |
| Dehalobacterium_f | 0.1±0.1 | 0.2±0.1 | 0.2±0.2 | 0.2±0.1 |
| Coriobacteriaceae | 0.1±0.1 | 0.1±0.0 | 0.1±0.0 | 0.1±0.0 |
| Erysipelotrichaceae | 0.1±0.1 | 0.0±0.0 | 0.0±0.0 | 0.1±0.0 |
| Sutterellaceae | 0.1±0.1 | 0.0±0.0 | 0.0±0.0 | 0.2±0.1**^#^** |
| Mogibacterium_f | 0.1±0.0 | 0.1±0.0 | 0.1±0.0 | 0.1±0.0**^#^** |
| PAC000197_f | 0.0±0.0 | 0.0±0.0 | 0.1±0.1 | 0.2±0.1 |
| FR888536_f | 0.0±0.0 | 0.1±0.1 | 0.2±0.2 | 0.6±0.6 |
| Deferribacteraceae | 0.0±0.0 | 0.3±0.5 | 0.0±0.0 | 0.0±0.0 |
| Akkermansiaceae | 0.0±0.1 | 1.3±3.4 | 1.1±1.0 | 0.3±0.2 |

Values indicate mean± SD. ******p*<0.05 vs NC and **^#^***p*<0.05 vs IS.

Table S6. Effects of Rd and CK on the gut microbiota composition (at a genus level) in mice treated with IS

|  | Composition (%) | | | |
| --- | --- | --- | --- | --- |
|  | NC | IS | ID1 | IC1 |
| Lactobacillus | 31.5±9.8 | 9.3±6.9***** | 0.8±0.4**^#^** | 3.0±2.7**^#^** |
| PAC000186_g | 10.2±2.8 | 7.53.4 | 10.4±3.8 | 8.2±3.6 |
| PAC001068_g | 7.5±2.2 | 6.9±3.1 | 7.3±2.0 | 10.4±3.5 |
| Bacteroides | 4.6±1.1 | 3.4±2.3 | 5.9±3.8 | 4.0±2.7 |
| KE159538_g | 4.2±3.5 | 5.0±6.6 | 1.8±1.1 | 2.2±2.7 |
| PAC001074_g | 2.9±2.4 | 1.6±1.3 | 2.4±1.3 | 3.6±1.1**^#^** |
| Prevotella | 2.5±1.6 | 2.3±2.2 | 4.1±4.0 | 4.8±2.4 |
| PAC000198_g | 2.3±0.4 | 2.8±0.9 | 2.7±1.0 | 3.2±1.3 |
| Alistipes | 2.3±1.5 | 3.3±2.3 | 2.4±1.0 | 1.6±1.6 |
| PAC001127_g | 1.9±0.8 | 1.3±0.9 | 1.6±0.6 | 1.1±0.7 |
| Helicobacter | 1.8±0.7 | 7.2±4.9***** | 1.9±1.8**^#^** | 2.3±1.9 |
| Alloprevotella | 1.4±2.1 | 1.5±1.3 | 4.0±3.6 | 2.7±2.0 |
| PAC001124_g | 1.2±1.7 | 0.9±0.9 | 0.8±0.8 | 1.3±0.9 |
| Muribaculum | 1.1±0.6 | 1.4±0.9 | 1.9±0.5 | 3.5±1.7 |
| PAC000664_g | 1.1±1.4 | 2.4±1.6 | 2.1±1.5 | 2.0±1.5 |
| PAC001066_g | 1.1±0.4 | 0.9±0.7 | 1.8±0.8**^#^** | 0.4±0.5 |
| Odoribacter | 1.0±1.4 | 0.5±0.7 | 0.1±0.1 | 0.0±0.0 |
| PAC001765_g | 1.0±0.6 | 1.1±0.4 | 1.3±0.5 | 1.0±0.3 |
| PAC001112_g | 0.9±0.3 | 1.6±1.3 | 0.9±0.3 | 1.8±0.5 |
| Paraprevotella | 0.9±0.9 | 2.3±2.6 | 2.6±1.8 | 4.7±5.0 |
| Arthromitus | 0.9±1.2 | 0.1±0.1 | 0.1±0.1 | 0.1±0.0 |
| Pseudoflavonifractor | 0.9±0.6 | 1.6±0.6***** | 1.8±1.4 | 1.3±0.5 |
| Prevotellaceae_uc | 0.9±0.7 | 3.1±4.9 | 4.7±3.7 | 4.9±4.3 |
| Oscillibacter | 0.7±0.8 | 2.3±1.3 | 1.8±1.1 | 1.1±0.5**^#^** |
| PAC001512_g | 0.7±0.6 | 1.0±0.6 | 2.9±1.4**^#^** | 2.3±1.8 |

Values indicate mean± SD. ******p*<0.05 vs NC and **^#^***p*<0.05 vs IS.

Table S7. Effects of RG, fRG, Rd, and CK on the gut microbiota composition (at a phylum level) in mice treated with UCDF

| Taxon Name | Composition (%) | | | | | |
| --- | --- | --- | --- | --- | --- | --- |
|  | NC | FT | FF50 | FR50 | FD1 | FC1 |
| Bacteroidetes | 49.0±18.8 | 54.8±15.5 | 59.2±7.9 | 59.9±15.3 | 54.6±27.8 | 57.4±12.4 |
| Firmicutes | 47.5±18.9 | 39.8±15.1 | 35.2±7.5 | 35.3±13.9 | 39.9±25.5 | 37.6±9.8 |
| Proteobacteria | 2.4±1.5 | 3.7±1.8 | 3.5±1.2 | 3.0±1.6 | 4.4±2.8 | 4.0±3.8 |
| Tenericutes | 0.6±0.9 | 0.7±0.5 | 0.3±0.0 | 0.7±0.4 | 0.6±0.4 | 0.5±0.2 |
| Actinobacteria | 0.3±0.2 | 0.5±0.6 | 0.3±0.1 | 0.4±0.2 | 0.2±0.1 | 0.3±0.1 |
| Cyanobacteria | 0.1±0.1 | 0.3±0.2 | 1.3±2.0 | 0.1±0.1 | 0.3±0.3 | 0.1±0.0 |
| Deferribacteres | 0.0±0.1 | 0.2±0.2 | 0.1±0.1 | 0.3±0.4 | 0.0±0.0 | 0.1±0.0 |
| Verrucomicrobia | 0.0±0.0 | 0.1±0.1 | 0.1±0.1 | 0.3±0.3 | 0.0±0.0 | 0.0±0.0 |
| Saccharibacteria_TM7 | 0.0±0.0 | 0.0±0.0 | 0.0±0.0 | 0.0±0.0 | 0.0±0.0 | 0.0±0.0 |
| Planctomycetes | 0.0±0.0 | 0.0±0.0 | 0.0±0.0 | 0.0±0.0 | 0.0±0.0 | 0.0±0.0 |

Values indicate mean± SD. ******p*<0.05 vs NC and **^#^***p*<0.05 vs FT.

Table S8. Effects of fRG, Rd, and CK on the gut microbiota composition (at a family level) in mice treated with UCDF

| Taxon Name | Composition (%) | | | | | |
| --- | --- | --- | --- | --- | --- | --- |
|  | NC | FT | FF50 | FR50 | FD1 | FC1 |
| Muribaculaceae | 31.0±9.4 | 33.8±11.1 | 39.1±9.2 | 36.4±13.4 | 29.9±16.7 | 27.7±4.8 |
| Lactobacillaceae | 25.8±19.3 | 3.8±3.2***** | 0.8±0.4**^#^** | 1.9±2.7 | 0.5±0.3**^#^** | 2.5±4.7 |
| Lachnospiraceae | 15.8±7.7 | 27.0±16.6 | 22.9±7.3 | 25.1±12.5 | 30.3±23.3 | 23.9±9.7 |
| Prevotellaceae | 7.0±6.0 | 13.4±8.3 | 11.6±3.4 | 15.4±8.0 | 16.3±10.6 | 22.7±8.2 |
| Bacteroidaceae | 5.3±3.8 | 4.4±4.3 | 5.0±5.2 | 4.2±3.3 | 3.6±2.5 | 3.3±1.7 |
| Rikenellaceae | 4.4±3.6 | 2.4±1.1 | 2.2±1.3 | 3.0±1.4 | 3.4±1.1 | 3.0±1.0 |
| Ruminococcaceae | 4.2±2.1 | 8.0±3.3***** | 10.3±1.8 | 7.2±3.2 | 8.1±2.7 | 10.1±2.1 |
| Helicobacteraceae | 1.8±1.3 | 2.6±1.8 | 1.7±0.5 | 1.7±1.3 | 2.8±3.2 | 3.0±3.7 |
| Odoribacteraceae | 0.9±1.3 | 0.3±0.2 | 0.4±0.3 | 0.3±0.2 | 0.6±0.4 | 0.1±0.1 |
| Clostridiaceae | 0.7±0.9 | 0.2±0.1 | 0.1±0.1 | 0.1±0.1 | 0.3±0.3 | 0.1±0.1 |
| Desulfovibrionaceae | 0.4±0.2 | 0.6±0.3 | 1.3±0.9 | 0.9±0.4 | 0.9±0.4 | 0.7±0.3 |
| Mycoplasmataceae | 0.4±1.0 | 0.4±0.5 | 0.0±0.0 | 0.3±0.4 | 0.3±0.4 | 0.3±0.1 |
| Enterococcaceae | 0.3±0.4 | 0.1±0.1 | 0.1±0.1 | 0.1±0.1 | 0.0±0.0**^#^** | 0.0±0.0 |
| Christensenellaceae | 0.3±0.2 | 0.2±0.1 | 0.3±0.2 | 0.3±0.0**^#^** | 0.2±0.1 | 0.3±0.2 |
| Porphyromonadaceae | 0.2±0.1 | 0.3±0.3 | 0.3±0.1 | 0.3±0.1 | 0.4±0.3 | 0.2±0.1 |
| AC160630_f | 0.2±0.2 | 0.2±0.1 | 0.7±0.2**^#^** | 0.4±0.3 | 0.3±0.2 | 0.4±0.2 |
| Coriobacteriaceae | 0.2±0.1 | 0.4±0.6 | 0.3±0.1 | 0.4±0.2 | 0.2±0.1 | 0.2±0.1 |
| Erysipelotrichaceae | 0.2±0.1 | 0.2±0.2 | 0.2±0.2 | 0.2±0.1 | 0.1±0.0 | 0.1±0.2 |
| PAC000197_f | 0.1±0.1 | 0.1±0.1 | 0.2±0.0 | 0.2±0.2 | 0.2±0.1 | 0.1±0.1 |
| FR888536_f | 0.1±0.1 | 0.3±0.2 | 1.3±2.0 | 0.1±0.1 | 0.3±0.3 | 0.1±0.0 |
| Dehalobacterium_f | 0.1±0.1 | 0.2±0.2 | 0.3±0.2 | 0.3±0.2 | 0.2±0.1 | 0.3±0.1 |
| Sutterellaceae | 0.1±0.1 | 0.1±0.1 | 0.1±0.0 | 0.1±0.0 | 0.1±0.1 | 0.1±0.0 |
| Bifidobacteriaceae | 0.1±0.2 | 0.1±0.1 | 0.0±0.0 | 0.0±0.1 | 0.0±0.0 | 0.0±0.0 |
| Mogibacterium_f | 0.1±0.0 | 0.1±0.0 | 0.1±0.0 | 0.1±0.0 | 0.1±0.0 | 0.1±0.0 |
| Rhodospirillaceae | 0.0±0.1 | 0.3±0.5 | 0.3±0.2 | 0.3±0.4 | 0.5±0.6 | 0.2±0.1 |

Values indicate mean± SD. ******p*<0.05 vs NC and **^#^***p*<0.05 vs FT.

Table S9. Effects of fRG, Rd, and CK on the gut microbiota composition (at a genus level) in mice treated with UCDF

| Taxon Name | Composition (%) | | | | | |
| --- | --- | --- | --- | --- | --- | --- |
|  | NC | FT | FF50 | FR50 | FD1 | FC1 |
| Lactobacillus | 25.7±19.2 | 3.8±3.1***** | 0.8±0.4**^#^** | 1.9±2.7 | 0.5±0.3**^#^** | 2.5±4.6 |
| PAC000186_g | 8.5±2.6 | 8.4±4.2 | 7.1±1.8 | 8.0±2.7 | 5.9±2.2 | 6.5±1.5 |
| PAC001068_g | 7.9±2.2 | 9.7±5.7 | 10.6±5.0 | 8.5±3.3 | 8.9±7.4 | 6.4±1.9 |
| Bacteroides | 5.3±3.8 | 4.4±4.3 | 5.0±5.1 | 4.2±3.3 | 3.6±2.5 | 3.3±1.7 |
| Alistipes | 3.8±3.3 | 1.8±1.0 | 1.8±1.2 | 2.5±1.6 | 2.9±1.0 | 2.6±1.1 |
| KE159538_g | 3.4±3.0 | 5.6±8.1 | 1.1±0.5 | 2.1±1.9 | 1.1±1.1 | 1.9±2.1 |
| Prevotellaceae_uc | 3.4±4.8 | 8.1±6.7 | 5.6±2.6 | 6.8±4.7 | 10.3±7.2 | 14.0±4.4 |
| PAC000198_g | 3.1±2.3 | 3.7±2.3 | 4.1±2.1 | 3.2±3.1 | 3.1±1.9 | 3.2±0.8 |
| PAC001074_g | 2.3±1.1 | 2.0±0.9 | 2.3±0.9 | 1.7±1.2 | 2.0±1.6 | 1.2±0.9 |
| Prevotella | 2.1±1.9 | 3.5±2.2 | 5.1±2.2 | 4.1±2.8 | 4.6±3.8 | 5.6±2.0 |
| Helicobacter | 1.8±1.3 | 2.6±1.8 | 1.7±0.5 | 1.7±1.3 | 2.8±3.2 | 3.0±3.7 |
| Muribaculum | 1.6±1.2 | 1.6±0.6 | 3.8±2.6 | 4.8±2.5 | 1.5±1.3 | 1.3±0.8 |
| PAC001124_g | 1.3±1.2 | 0.8±0.9 | 0.3±0.1 | 0.5±0.7 | 1.8±2.4 | 0.3±0.6 |
| PAC001127_g | 1.2±0.6 | 0.7±0.4 | 1.2±0.5 | 1.6±1.3 | 2.2±1.5 | 1.0±0.5 |
| PAC001512_g | 1.2±1.0 | 1.2±0.9 | 0.2±0.1 | 0.8±0.5 | 0.4±0.2 | 1.4±0.5 |
| PAC001112_g | 1.1±0.5 | 1.3±0.4 | 3.3±2.1 | 1.4±0.4 | 1.0±0.5 | 1.3±0.5 |
| PAC000664_g | 1.0±0.8 | 2.5±1.5 | 1.7±0.5 | 3.2±2.0 | 2.6±0.9 | 2.2±1.3 |
| Pseudoflavonifractor | 1.0±0.6 | 1.8±1.1 | 1.9±0.6 | 1.7±0.9 | 1.6±1.1 | 1.7±0.6 |
| PAC000661_g | 1.0±1.0 | 0.9±0.8 | 1.6±0.8 | 0.9±0.4 | 0.9±0.5 | 1.3±1.3 |
| PAC001066_g | 0.9±0.5 | 0.7±0.5 | 0.2±0.1 | 1.1±0.7 | 0.2±0.1**^#^** | 0.6±0.3 |
| PAC001765_g | 0.9±0.5 | 1.2±0.6 | 1.0±1.0 | 1.0±0.7 | 1.2±0.6 | 0.8±0.3 |
| Odoribacter | 0.9±1.3 | 0.2±0.3 | 0.3±0.3 | 0.2±0.2 | 0.5±0.5 | 0.1±0.1 |
| Eubacterium_g6 | 0.9±0.4 | 1.0±1.3 | 2.3±4.3 | 3.0±4.9 | 2.4±2.7 | 2.6±3.2 |
| Oscillibacter | 0.8±0.8 | 1.2±0.8 | 1.7±1.4 | 1.2±0.6 | 1.7±1.5 | 1.7±1.0 |
| Paraprevotella | 0.8±0.9 | 1.3±1.4 | 0.3±0.4 | 2.3±3.3 | 1.4±1.9 | 3.1±4.9 |

Values indicate mean± SD. ******p*<0.05 vs NC and **^#^***p*<0.05 vs FT.
